# Effect of Season and High Ambient Temperature on Activity Levels and Patterns of Grizzly Bears (*Ursus arctos*)

**DOI:** 10.1371/journal.pone.0117734

**Published:** 2015-02-18

**Authors:** Michelle L. McLellan, Bruce N. McLellan

**Affiliations:** 1 South Coast Grizzly Bear Project, Nelson, British Columbia, Canada; 2 British Columbia Fish, Wildlife and Habitat Management, D’Arcy, British Columbia, Canada; Sonoma State University, UNITED STATES

## Abstract

Understanding factors that influence daily and annual activity patterns of a species provides insights to challenges facing individuals, particularly when climate shifts, and thus is important in conservation. Using GPS collars with dual-axis motion sensors that recorded the number of switches every 5 minutes we tested the hypotheses: 1. Grizzly bears (*Ursus arctos*) increase daily activity levels and active bout lengths when they forage on berries, the major high-energy food in this ecosystem, and 2. Grizzly bears become less active and more nocturnal when ambient temperature exceeds 20°C. We found support for hypothesis 1 with both male and female bears being active from 0.7 to 2.8 h longer in the berry season than in other seasons. Our prediction under hypothesis 2 was not supported. When bears foraged on berries on a dry, open mountainside, there was no relationship between daily maximum temperature (which varied from 20.4 to 40.1°C) and the total amount of time bears were active, and no difference in activity levels during day or night between warm (20.4–27.3°C) and hot (27.9–40.1°C) days. Our results highlight the strong influence that food acquisition has on activity levels and patterns of grizzly bears and is a challenge to the heat dissipation limitation theory.

## Introduction

Circadian rhythms are fundamental to most organisms and are driven by endogenous processes that are themselves influenced largely by daily patterns of light and darkness. These rhythms are assumed to enhance the survival and reproductive fitness of individuals [1]. For mammals, circadian rhythms are likely timed to enable efficient foraging and digestion while reducing the risk of predation but they may also be constrained by thermoregulatory requirements and therefore by climate. Daily activity patterns of mammals are strongly influenced by circadian rhythms but these patterns may vary with the foods available, social/mating behaviour, perceived predation risk and short-term weather events [2–5]. Understanding factors that influence activity patterns of a species may help us understand critical challenges facing individuals, particularly when climate shifts, and thus may be important in conservation management of populations at high risk of extirpation [6].

Bears are large carnivores and in many areas have no predators except other bears and people. When far from people, the type and abundance of food are thought to influence their daily activity patterns and overall activity levels. During winter, bears living in temperate ecosystems hibernate and therefore must ingest sufficient nutrients in about 7 months to sustain them for the entire year. In the summer and autumn, temperate bears become hyperphagic [7] while foraging on high-energy foods and they rapidly deposit fat needed for hibernation [8,9]. Increased activity when high-energy summer or autumn foods such as berries or nuts were available has been reported for American black bears [2,10,11] as well as Asiatic black bears that hibernated [12] and a tropical population that did not hibernate [6]. Although lower levels of activity have been reported for grizzly bears early in the spring and late in the autumn than during summer [13,14], an increase in activity during periods of hyperphagia has not been clearly shown for this species and increases of activity during summer may be confounded with longer day length [15].

In addition to bears increasing the amount of time they are active when they are rapidly depositing fat while feeding on high-energy foods, it is probable that foraging bout lengths should vary depending on the type of food eaten. In some regions, small and dispersed (i.e. not bunched) fruits such as huckleberries are critical foods when bears deposit fat in summer and autumn [9]. These fruits are also highly digestible so bears > 80 kg in mass have a difficult time ingesting them quickly enough for maximum fat deposition [16,17]. Bears feeding on fruits likely have longer active bout lengths than when feeding on other foods as they try to fill their guts on small, dispersed fruits.

Weather, and in particular high temperature, likely has a strong effect on activity levels and daily activity patterns of mammals [2,4,5,13,18]. Indeed, mammals appear to spend their lives shifting between times when energy intake is limited to times when their ability to use energy is constrained by their ability to dissipate body heat [18]. Bears living in temperate ecosystems may face these contrasting demands more than most other species. They are large bodied endotherms with relatively short, powerful limbs and do not have sweat glands so are not well suited for heat dissipation. Yet, bears must develop thick under-fur and deposit a substantial layer of fat needed for months of hibernation. Consequently, hot summer and autumn weather should constrain activity. Black bears reduced activity when temperatures exceeded 23°C [2] and grizzly bears reduced activity at 20°C [13]. Although predicted climate changes may affect grizzly bear populations in numerous and complex ways, direct tolerance of higher temperatures is likely important if heat dissipation is a major constraint [18]. This constraint would be particularly important in southern areas where bears deposit fat foraging on fruits that grow best in open areas with little or no shade [19].

We studied the activity patterns of grizzly bears with GPS collars equipped with activity sensors in small (<50 bears), endangered populations in southwestern British Columbia. We first tested hypotheses regarding factors that have been proposed to influence grizzly bear activity while accounting for changing day length. In particular, we compared activity levels by season to test if bears increase activity and active bout lengths when they forage on berries, the major high-energy food in this ecosystem during summer when they deposit fat needed for hibernation [9]. We then tested the hypothesis that bears become less active and more nocturnal as daytime temperatures increase. Our study was located in one of the hottest areas in Canada with or without grizzly bears; temperatures sometimes exceeded 40°C so is was well suited to investigate the direct implications of warming climate.

## Methods

### Study area

We studied grizzly bear activity budgets in a 4000 km^2^ area in the eastern portion of the Coast Mountain Range in southwestern British Columbia (50.6°N, 122.5°W). These are rugged mountains with elevations in the study area ranging from 240 m to 2920 m. Air masses moving eastward from the Pacific Ocean dominate the climate and result in temperate rain forests on the west side of the mountain range but conditions become increasing dry towards the rain shadow in the eastern mountains where we studied bears. Near the lowest elevation in the study area, the 25-year average daily maximum and minimum temperatures were 28.2°C and 11.6°C in July and 0.6°C and -2.9°C respectively in January. Annual precipitation at this elevation averaged 500 mm. In the mountains at 1830 m, the average daily maximum and minimum July temperatures were 17.0 and 5.3°C and in January these were -3.5 and -9.5°C. In the mountains on the dry side of the study area, an average of 730 mm of precipitation fell.

Vegetation in the study area reflects the influence of high mountains, the transition of precipitation, and natural plus human-caused disturbances. Douglas fir (*Pseudotsuga menziesii*) and ponderosa pine (*Pinus ponderosa*) are the most common conifers at lower elevations with cottonwood (*Populus trichocarpa*) and western red cedar (*Thuja plicata*) along streams. At mid elevation, forests of Engelmann spruce (*Picea engelmannii*) and subalpine fir (*Abies lasiocarpa*) are most common but these are often fragmented by lush avalanche chutes as well as numerous clear cuts. At higher elevations, subalpine fir grows in clumps which are often stunted forming krummholtz within extensive alpine meadows. Mountain peaks are rock and often rise far above the alpine meadows. There are only a few small glaciers in the study area.

We investigated the implications of hot weather on activity levels and patterns of grizzly bears in a 5 km^2^, relatively low-elevation (300 m to 1400m) area called Anderson Shoulder. This area was burned by wildfire on 24 July, 1958. The area is steep and dry on a southeast (150°) aspect and very few conifers have regenerated to provide shade but there is an abundance of Saskatoon berries (*Amelanchier alnifolia*). Four transmission lines cross this area ensuring permanent early-seral conditions.

### Data collection

Grizzly bears were captured primarily by darting from a helicopter but one was captured using a foot snare. Bears were chemically immobilized using a tiletamine and zolazepam combination using an average dosage of 11mg/kg administered by a Pneu-dart internal charge dart gun remote delivery system. The IACUC of British Columbia is the Animal Care Committee of the British Columbia Fish, Wildlife, and Habitat Management Branch. All capture, immobilization and handling methods were approved by this body. Field permits were granted by the British Columbia Ministry of Environment and British Columbia Fish, Wildlife, and Habitat Management Branch. Once immobilized, bears were fitted with GPS collars (4400S and 4400M Lotek Wireless Inc., Newmarket, Ontario, Canada). These collars had pre-timed release mechanisms but we also inserted a canvas spacer to ensure collars came off if the release mechanisms failed. The collars were programmed to only transmit a VHF beacon and to record a GPS location when bears were not hibernating. The 4400S collars were programmed to attempt to locate the bear every 3 hours while the 4400M collars attempted to record its location every hour. Collars were tested for location accuracy prior to deployment. Both models contained two perpendicular, captive-ball tip switches that were equally sensitive to up and down and side to side movements. The number of times that each sensor was triggered (maximum = 255) was recorded every 5 minutes and stored in memory. We downloaded all the activity data once the collar was retrieved from the bear. We summed the number of the two sensors so the resultant 5 minute period could vary from 0 to 510 sensor switches. We did not use data collected during the first week after capture to ensure immobilization no longer influenced activity.

Others [20] have used the same brand and model of collar on Asiatic black bears (*U. thibetanus*). By observing videos of 2 bears equipped with these collars they determined that that 0 to 41 switches of both sensors combined per 5 minute period indicated inactivity while >42 always indicated an active bear. We tested whether 41 switches also correctly categorized grizzly bear activity by plotting the frequency distribution of the number of switches per 5 minute period for each bear and predicted that 0 and very few switches per period (< 41) would frequently be recorded and reflect an inactive bear [20]. There would then be very rare occurrences near 41 switches because this is too many for an inactive bear but too few for an active bear. These infrequent numbers of switches would then be followed by a normal distribution of switches with a means of > 100 reflecting active bears [20]. The optimal cut-point for our sample of bears would be where there the minimum occurrence of switches between the inactive and active distributions.

In addition to the amount of time per day that bears were active or inactive, we were also interested in the duration of active and inactive bouts. Determining bout length from the number of switches in a collar every 5 minutes becomes more complicated because long periods of inactivity, for example, may be fragmented by very short periods of apparent activity that was likely caused by the bear shifting body position, lifting their head to look around [12], or a minor irritant such as a flying insect. To quantify actual switch patterns of collars on active and inactive bears we visited grizzly bear GPS locations on the ground shortly after the bear had moved away and searched for sign of fresh bedding, feeding or travelling. We extracted the activity data for periods of time when our field check only found sign of bedding and the GPS locations were in the same location for 2 or more consecutive hours to investigate inactive data patterns. Alternatively, sites with no sign of bedding and moving locations were used to inspect the pattern of active bears. We scanned these data of inactive and active bouts and found many consecutive 5 minute periods with 0–10 switches for inactive bears and > 50 switches for active bears but these continuous sequences were sometimes interrupted by 1 to 3 periods (5 to 15 minutes) of the opposite pattern. We changed these 1 to 3 periods to be consistent with the long bout pattern that bounded them.

To test the hypothesis that bears would increase their level of activity and would have longer active bout lengths when they were foraging on fruit, we divided the active year into 3 seasons that is standard with bear populations that forage primarily on berries in the summer [19,21,22]. The spring or herb-bulb season was from when all bears had emerged from hibernation and moved away from their den sites to when they began to feed on berries. We were interested in activity levels and patterns associated with spring-time foraging and not activity associated with den emergence so we only used data collected after 22 May, which was the latest date a bear moved from its den location. The summer or berry season was when bears forage primarily on Saskatoon berries and black huckleberry (*Vaccinium membranaceum*) and was delineated for each individual and each year depending on when they first moved to berry fields for more than one day and when they left berry fields in the autumn. The beginning of the berry season for individuals varied from July 6 to August 25 and this season ended between September 7 and October 12. For the autumn or post berry season, we did not use activity data after 22 October because that was the earliest that a bear moved close to where it eventually denned and we wanted to avoid mixing pre-denning activity in the post berry foraging season. We also excluded data from bears for entire days when one or more of their locations were within 500 m of human settlement [21] because developed sites may effect activity [13].

The level of activity each season is potentially confounded by the amount of daylight, therefore we extracted nautical and civil twilight times for each day for the center of our study area from the National Research Council website (http://www.nrc-cnrc.gc.ca/eng/services/sunrise/advanced.html accessed June, 2013). We used each week as the sampling unit in a linear mixed effects model with the individual bear as the random effect. The dependent variable was either the average number of hours per day that each bear was active each week or the average active and inactive bout lengths each week. The independent variables were the average number of hours between civil twilights per day each week, sex, and season. Activity data were excluded for weeks that overlapped seasonal boundaries. We used R [23] and lmerTest package [24] for these analyses.

To describe the daily activity patterns of bears and to test if these patterns changed by season and sex we categorized each 5 minute activity sample into 1 of 6 daily periods: day (sunrise to sunset), evening twilight (sunset to end of civil twilight), evening nautical (end of civil to end of nautical twilight), night (end of evening nautical to start of morning nautical twilight), morning nautical (start of morning nautical twilight to start of civil twilight), and morning twilight (start of civil twilight to sunrise). We used a mixed effects model with the individual bear being the random intercept, the period of day, season, and sex of the bear being fixed effects, and the dependent variable being the arcsine of the average proportion of time that bears were active during each daily period. To test the hypothesis that bears would reduce their level of activity as temperatures increased we used the arcsine of the proportion of the day that each bear was active as the dependent variable and the daily maximum temperature as the independent variable in a linear regression. We used bear activity data from all the days that bears were found in a low-elevation Anderson Shoulder burn where accurate temperature data were available from the BC Hydro Shalalth weather station (http://tools.pacificclimate.org/dataportal/pcds_map/, station number 2504). This station is on the same aspect, within 100m elevation, and 8km from the center of the bear locations within Anderson Shoulder burn. To test the hypothesis that bears would become less diurnal and more nocturnal on hotter days, we first sorted all days that bears were found in the low-elevation burn by the maximum daily temperature and made 2 categories with an equal number of samples; warm days (daily maximum between 20.4 and 27.3°C) and hot days (between 27.9 and 40.1°C). We compared the proportion of time each bear was active (arcsine transformed) each day during the same 2 daily periods with civil twilights pooled with day and nautical twilights pooled with night using a linear mixed effects model with the individual bear as the random effect.

## Results

We were able to retrieve and download 385 and 200 complete weeks (1.12*10^6^ samples) of activity data excluding hibernation and capture periods from 10 female and 9 male grizzly bears, respectively. The frequency plot of the number of switches per 5 minute sampling period for each bear, as expected, had a peak at 0 switches from inactive bears and a second, broader peak that varied among individuals from about 90 to 190 switches reflecting active bears. Averaged over bears, the minimum point between these two peaks was at 36 switches which we used to separate inactive from active bears. To examine data patterns of active and inactive bears in the field we visited 66 sites on the ground where bedding was the only bear sign observed and 141 sites where no sign of bedding was found. We extracted the activity data from 6147 sampling periods from when bears were at these beds and 11,452 sample periods when they were active. Of these inactive bouts, 23 (35%) were interrupted with 1 period with > 36 switches, 6 (9%) had 2 consecutive periods with > 36 switches and 4 (6%) were interrupted by 3 periods with > 36 switches. Changing these 47 samples of apparent activity to inactive when they were bounded by > 1 hour of consistent inactivity increased the total time inactive by only 0.8% but in doing so stopped fragmenting long inactive bouts and adding very short (5–15 m) active bouts. Similarly, 33, 5, and 8 of the 141 active bouts had breaks of 1, 2, and 3 sampling periods, respectively, with < 36 switches. When these apparent inactive periods were changed to active, it increased the overall level of activity by 0.6%. All changes, therefore, decreased the raw measure of activity by only 0.12% but stopped the fragmentation of longer bouts. We changed all 1, 2 or 3 consecutive samples within long bouts when bounded before, after or both with an hour of the opposite pattern. Only 2 collared bears, both adult males, were found near human residences and therefore we removed 57 days of data from our analyses.

**Fig 1:**
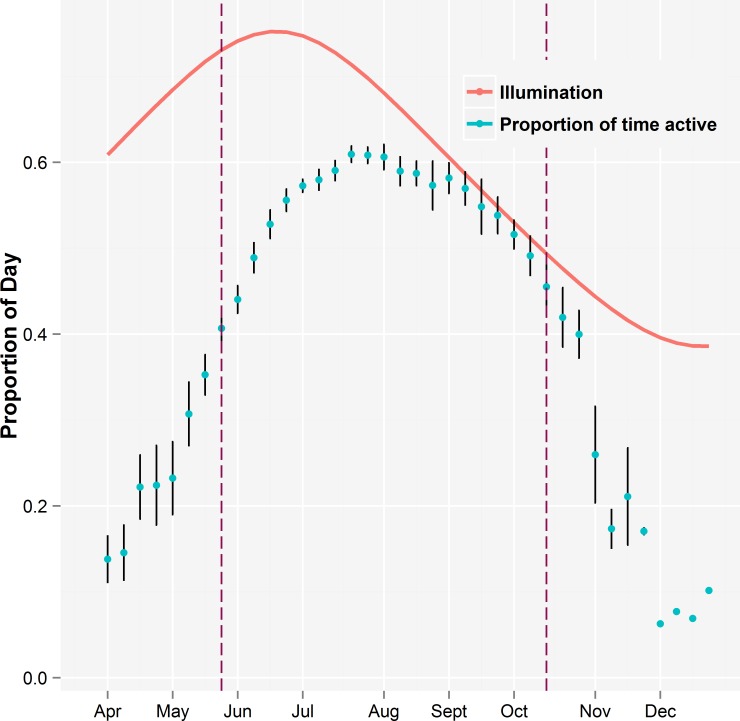
The proportion of time (± standard error) that individual grizzly bears were active during each week of the year. The sampling unit was the average for each bear each week. The vertical purple dashed lines were when all bears had moved away from denning areas in spring and before any bears had moved to their denning areas in autumn. Data collected between these times were used in the analyses. The solid curved red line shows the proportion of the day illuminated from the beginning of morning civil twilight to the end of evening civil twilight.

The average amount of time per day that bears were active was low early in the spring when bears had recently emerged from their dens but steadily increased until mid-July (Fig. 1). Bears maintained this high level of activity until September and then their level of activity steadily declined until they were approaching time for hibernation. Activity levels of bears that were not denned were very low in April, November, and December.

**Fig 2:**
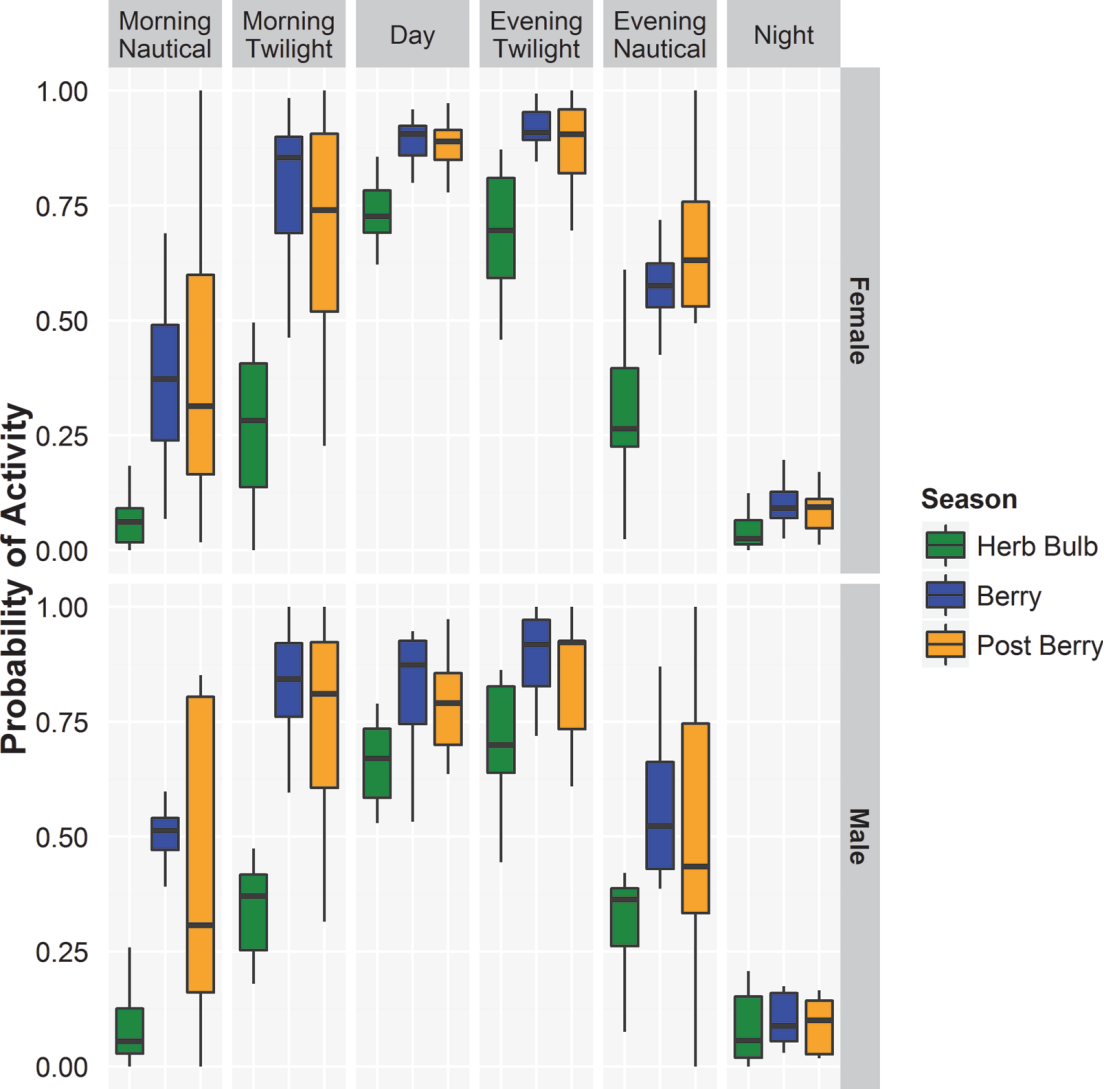
Proportion of time that female and male grizzly bears were active during different periods of the day during each season. Box plots show median and quartiles.

The hypothesis that bears increased their level of activity and duration of active bouts when foraging on berries was supported. Females were active an average of 14.75 ± 0.13 h d^-1^ and males 13.57 ± 0.40 h d^-1^ during the berry season and the mixed effects analysis suggested that this was greater (β = 1.52, *SE* = 0.37, *p* < 0.001) than the 13.41 ± 0.15 h d^-1^ and 12.84 ± 0.19 h d^-1^ for female and male bears respectively in the herb-bulb season even when all data before 22 May were excluded. Similarly, berry season activity levels were higher (β = 2.23, *SE* = 0.37, *p* < 0.001) than the 12.64 ± 0.24 h d^-1^ and 10.78 ± 0.62 h d^-1^ for female and male bears, respectively, during the post berry season even when data after 22 October were excluded (Table 1, Fig. 2). This analysis also suggested that females were more active than males (β = 1.55, *SE* = 0.53, *p* = 0.005) but there was a significant interaction between sex and the herb-bulb season (β = 1.11, *SE* = 0.46, *p* = 0.016) when the differences in activity between males and females was less than during the other two seasons. The average amount of daylight per week was not a significant factor influencing activity levels throughout the year (β = -0.05, *SE* = 0.10, *p* = 0.603).

**Table 1:** Mean duration (h) (N, *SE*) of active and inactive bouts and mean total time (h) active per day each week during each season for individual female and male grizzly bears in the southern coast mountains of British Columbia, 2005 to 2012.

	Herb Bulb		Berry		Post Berry	
Type	Female	Male	Female	Male	Female	Male
Active Bout	4.04	3.60	6.83	5.49	5.80	4.26
	(197,0.10)	(104,0.12)	(74,0.26)	(37,0.36)	(58,0.25)	(19, 0.35)
Inactive Bout	3.19	3.13	4.28	4.22	5.21	5.22
	(198,0.06)	(104,0.07)	(73,0.12)	(36,0.16)	(58, 0.20)	(19, 0.29)
Hours Active	13.41	12.84	14.75	13.57	12.64	10.78
	(197,0.15)	(104,0.19)	(74,0.13)	(37,0.40)	(58,0.24)	(19,0.62)

Active bout lengths were different between female and male bears (β = 1.59, *SE* = 0.38, *p* < 0.001) and they were longer during the berry season than during the herb-bulb season (β = 1.38, *SE* = 0.29, *p*< 0.001; Table 1) or the post berry season (β = 2.18, *SE* = 0.31, *p* < 0.001). The amount of daylight was inversely related to the length of active bouts (β = -0.58, *SE* = 0.08, *p*< 0.001) with shorter bouts being in the herb-bulb season when day length was long compared to during summer and autumn. There was a significant interaction between sex and season with the average active bout length during the herb-bulb season being more similar for males and females than during the other two seasons (β = 1.16, *SE* = 0.37, *p* = 0.002). The average duration of inactive bouts appears to have increased from herb-bulb, through the berry season, and into the post berry season for both male and female bears but only the length of daylight was a significant, negatively correlated factor (β = -0.475, *SE* = 0.042, *p* < 0.001) with shorter inactive bouts when day length was longer.

**Fig 3:**
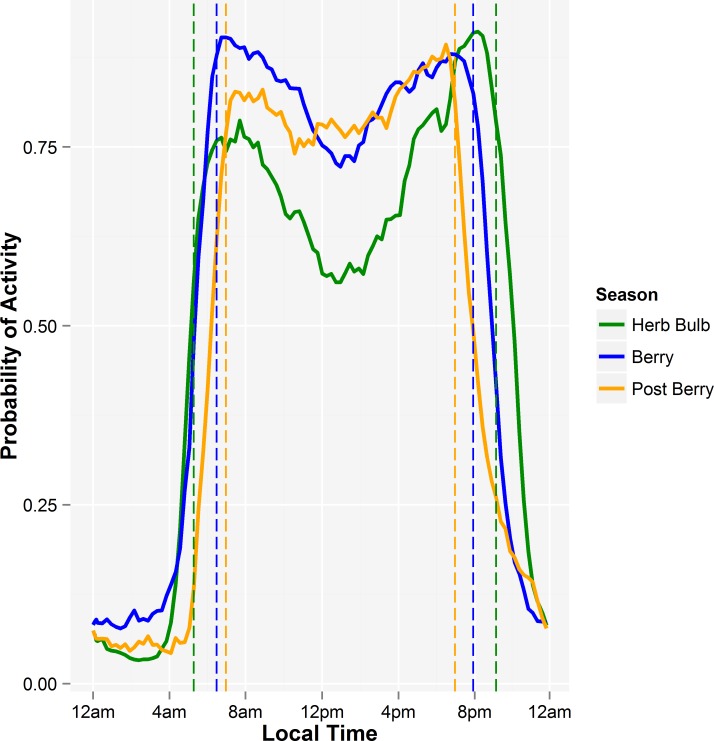
The average proportion of time that individual (male and female combined) grizzly bears were active during each hour during the spring herbbulb, summer berry, and autumn post berry seasons. The sampling unit was the average for each bear each hour. Vertical lines show the average sunrise and sunset for each season.

The mixed effects model with the individual bear being the random intercept, the period of day, season and sex of the bear being fixed effects, and the dependent variable being the arcsine of the proportion of time that bears were active showed activity levels differed by periods of day and season (both *p* < 0.001; Fig. 2). Bear activity levels were similar during the day and both civil twilight periods (β_MT_ = -0.16, *SE* = 0.09, *p* = 0.087, β_ET_ = 0.08, *SE* = 0.09, *p* = 0.375). Bears were less active at night (β_N_ = -1.00, *SE* = 0.09, *p* <0.001) and both nautical twilight periods (β_MN_ = -0.68, *SE* = 0.09, *p* <0.0001, β_EN_ = -0.45, *SE* = 0.09, *p* <0.001) than during the day (Fig. 2). The interaction between period and season was significant for night (β_HerbBulb-N_ = 0.23, *SE* = 0.17, *p* = 0.047) and morning twilight (β_HerbBulb-MT_ = -0.33, *SE* = 0.09, p <0.001) in herb-bulb season when bears were less active than would otherwise be predicted by season and period alone (Fig. 2). Both male and female bears were diurnal each season (Fig. 3) and were active, on average, 78.3% of the time between sunrise and sunset. Bears were also frequently active during the evening civil twilight period (77.3%), but activity was reduced during the nautical twilight (47.1%) before being very low at night (8.9%). In the mornings, particularly during the berry and post berry season, activity levels began to increase in the morning nautical twilight and continued increasing through the civil twilight period to the daylight period.

**Fig 4:**
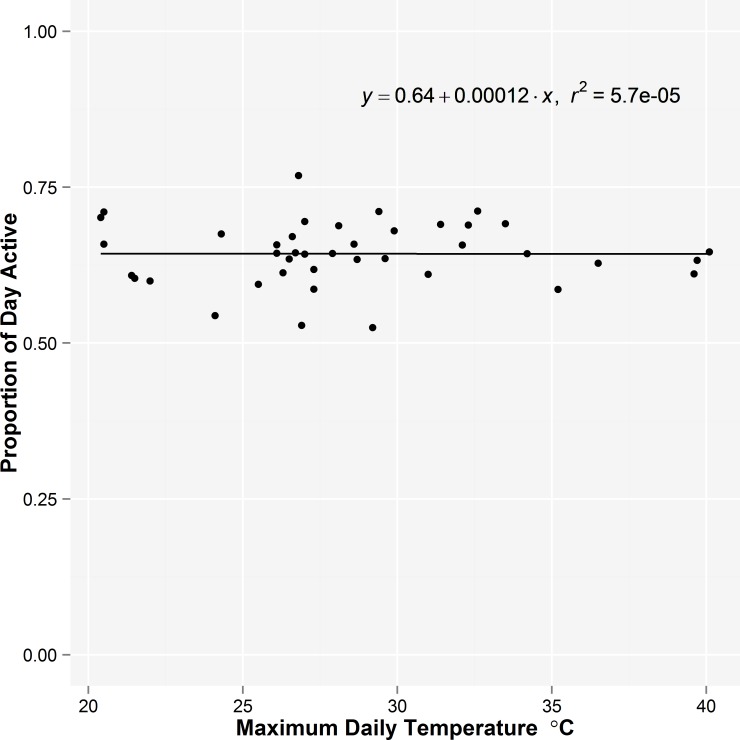
The maximum daily temperature measured in a Stevenson screen and the proportion of each 24 hour day that 3 female grizzly bears were active on the steep, southwest-facing Anderson Shoulder in July and August, 2006.

**Fig 5:**
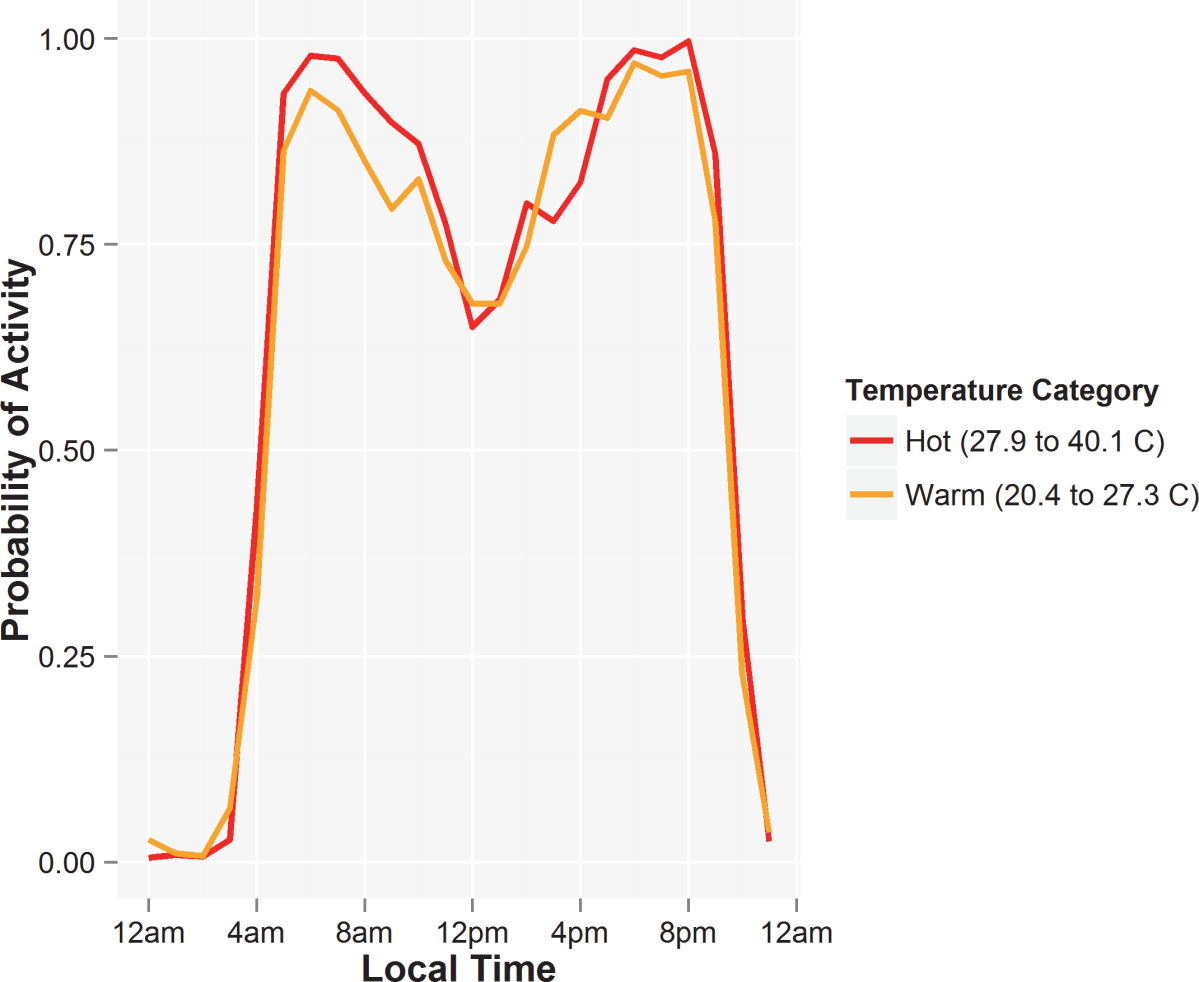
The average proportion of time that female grizzly bears were active during each hour on Anderson Shoulder on warm (daily maximum 20.4 to 27.3°C) and hot days (daily maximum 27.9 to 40.1°C) days between 6 July and 16 August 2006 where they feed mostly on Saskatoon berries.

We obtained a total of 97 complete days of activity data from 3 female bears when they fed on fruit on the largely unforested, Anderson Shoulder during a period when maximum daily temperatures in the shade varied between 20.4 to 40.1°C and daily minimums were 10.5 to 22.8°C. Two were lactating females with 3 and 2 yearlings and 1 was alone. There was no relationship between the daily maximum temperature and the total amount of time bears were active during each 24 hour period (*r* ^2^ = 0.001, *p* = 0.743; Fig. 4.). A mixed effect model with bear as the random intercept found no interaction between temperature category (daily maximums of 20.4–27.3 and 27.9–40.1) and level of activity during night and day (β = 0.043, *SE* = 0.042, *p* = 0.316; Fig. 5).

## Discussion

The hypothesis that bears would be most active and have longest active bouts when feeding on small, dispersed fruits was supported by our results. In regions where berries dominate their summer and autumn diet, bears deposit fat needed for hibernation while feeding on these foods that are high in carbohydrates [9]. Ingestion rate of dispersed berries is low so only small bears can accumulate fat near the physiological maximum [16]. The high daily activity level of bears feeding on berries is likely due to the importance of this food to their over-winter survival and reproduction. The longer active bouts also reflect the importance of berries but also their low ingestion rate and high digestibility compared to most other foods [25]; it takes considerable time for a bear to fill its gut on wild berries [16,17].

Grizzly bears in the Flathead drainage of southeastern British Columbia also forage on small, dispersed huckleberry and buffalo berries (*Shepherdia canadensis*) during summer when they rapidly deposit fat needed for hibernation [9]. Bears in this area were also more active in the berry season than other times of year [26], with levels of activity very similar to what we documented in the south-coastal mountains. Bears in arctic Canada were expected to be more active in the autumn when berries were ripe but activity levels did not vary among seasons [15]. The decreasing day length in the autumn in the arctic may have limited activity when berries were ripe but also caribou, ground squirrels, and other small mammals were likely more important foods in that ecosystem [15].

Across other bear species, the implication of food type and abundance on daily activity levels has been mixed. Asiatic black bears (*Ursus thibetanus*) in Taiwan and Japan increased activity levels when acorns, the main energy food in these areas, became abundant [6] but not in China when bears fed on berries or acorns [27]. Similarly, daily activity levels of American black bears in Quebec were found to be highest when they foraged on berries and nuts [11] but in Tennessee activity levels were highest during the June and July breeding season and declined thereafter [2]. The varied results from these few species may reflect the behavioural plasticity that has enabled the Ursid family to successfully inhabit a broad range of habitats from the high arctic to tropical rainforests to the Gobi desert.

The grizzly bears we monitored were diurnal during all seasons and they remained primarily active through the evening twilight period as well. They were largely inactive at night. This activity pattern is consistent with most studies across North America where there were few people [15,26,28–30] as well as for captive bears [31,32]. Being active during the day likely enhances foraging efficiency for most foods, and in particular small plants or portions of plants such as roots or berries [33]. The reliance of eyesight while foraging for roots was demonstrated when we captured an adult male grizzly bear in another study area that, years previously, had lost his right eye. The claws on his right front paw were 7 cm straight distance from toe tip to claw tip and finely pointed while claws on his left paw, the one he could see, were ground down and only 3.8 cm long (BN McLellan unpubl. data). Clearly, this bear dug roots with the paw he could see.

Nocturnal activity of grizzly bears has been reported for some bears when catching salmon in spawning streams [34], when feeding heavily on elk (*Cervus elaphus*) calves [14] or more generally for the largely carnivorous adult male bears in this same ecosystem [13]. Bears feeding on salmon at night caught more fish per hour than they did during daylight and suggested fish had poor evasive behaviour at night [34]. Similarly, grizzly bears were more efficient at killing elk calves at night than during the day [14]. Grizzly bears were crepuscular or diurnal before elk calves were born and later in the year when bears shifted their diet from elk calves to plant foods [14]. Captive bears switched from being diurnal to nocturnal when fed at night, but were relatively insensitive to experimental changes in photoperiods [32], further suggesting that food is the main entrainment for grizzly bear activity.

The female bears we monitored on Anderson Shoulder during July and August when they foraged on Saskatoon berries did not change their level of activity or daily activity patterns when temperatures increased above 30°C and even above 40°C. In addition to the high temperatures measured in the Stevenson screen, there was little shade on this steep, south-west facing slope. Although the GPS locations of these bears led us to one water source, it was too small for wallowing. None of the bears were located in or adjacent to the lake in the valley below. The actual level of heat stress that these bears endured far exceeded the 20°C and 23°C temperatures that that caused Yellowstone grizzly bears [13] or Tennessee black bears [2] to decrease activity.

The heat tolerance of these bears was surprising because even tropical mammals (although these were ruminants) of similar body size reduced activity levels at these temperatures [35–37]. These bears could have reduced their level of activity or become more nocturnal but their foraging efficiency would likely have suffered. Similarly, they could have remained high in the mountains where it was cooler and where cold streams and snow patches were abundant but there only herbs, grass, and bulbs, which have less digestible energy than berries [25,38], would be available. In this case the heat dissipation limit theory [18] was not supported. In addition to the high temperatures and lack of shade on Anderson Shoulder, two of the three female grizzly bears were lactating so heat dissipation was likely challenging [39], yet they chose to remain active to acquire high-energy resources from the environment rather than to focus on dissipating heat effectively. Although the high temperatures did not appear to affect their daily activities, we do not know if the bears were as efficient depositing fat at these temperatures. Bears, of course, are unique in that they are the only family of large mammal that hibernate, so acquiring high-energy resources when they are available is essential.

Our study, along with several others, highlights the significance that food plays in determining the activity levels and patterns of grizzly bears and some of the temporal decisions they made to obtain required nutrients. Appreciating the importance of food to their behaviour and population ecology [8,9] continues to be the basis for wise grizzly bear management. Our results also contribute to our understanding of how these large, stocky, and fur-covered mammals appear to tolerate hot conditions when there are sufficient foraging rewards. Such knowledge will become increasingly important towards animal conservation as climates change.

## Supporting Information

S1 Table Grizzly bear activity data (Part 1) for each bear, season and five minute interval excluding capture influenced times and when bears were < 500m from human settlement. (XLSX)

S2 Table Grizzly bear activity data (Part 2) for each bear, season and five minute interval excluding capture influenced times and when bears were < 500m from human settlement. (XLSX)
